# Quantification of hydrogen production by intestinal bacteria that are specifically dysregulated in Parkinson's disease

**DOI:** 10.1371/journal.pone.0208313

**Published:** 2018-12-26

**Authors:** Anzu Suzuki, Mikako Ito, Tomonori Hamaguchi, Hiroshi Mori, Yuka Takeda, Ryuko Baba, Takeshi Watanabe, Ken Kurokawa, Susumu Asakawa, Masaaki Hirayama, Kinji Ohno

**Affiliations:** 1 Division of Neurogenetics, Center for Neurological Diseases and Cancer, Nagoya University Graduate School of Medicine, Nagoya, Japan; 2 Department of Pathophysiological Laboratory Sciences, Nagoya University Graduate School of Medicine, Nagoya, Japan; 3 Genome Evolution Laboratory, Center for Information Biology, National Institute of Genetics, Mishima, Japan; 4 Laboratory of Soil Biology and Chemistry, Department of Biological Mechanisms and Functions, Nagoya University Graduate School of Bioagricultural Sciences, Nagoya, Japan; Wageningen Universiteit, NETHERLANDS

## Abstract

Oral administration of hydrogen water ameliorates Parkinson’s disease (PD) in rats, mice, and humans. We previously reported that the number of putative hydrogen-producing bacteria in intestinal microbiota is low in PD compared to controls. We also reported that the amount of hydrogen produced by ingestion of lactulose is low in PD patients. The decreased hydrogen production by intestinal microbiota may be associated with the development and progression of PD. We measured the amount of hydrogen production using gas chromatography by seven bacterial strains, which represented seven major intestinal bacterial groups/genera/species. *Blautia coccoides* and *Clostridium leptum* produced the largest amount of hydrogen. *Escherichia coli* and *Bacteroides fragilis* constituted the second group that produced hydrogen 34- to 93-fold lower than *B*. *coccoides*. *Bifidobacterium pseudocatenulatum* and *Atopobium parvulum* constituted the third group that produced hydrogen 559- to 2164-fold lower than *B*. *coccoides*. *Lactobacillus casei* produced no detectable hydrogen. Assuming that taxonomically neighboring strains have similar hydrogen production, we simulated hydrogen production using intestinal microbiota that we previously reported, and found that PD patients produce a 2.2-fold lower amount of intestinal hydrogen compared to controls. The lower amount of intestinal hydrogen production in PD was also simulated in cohorts of two other countries. The number of hydrogen-producing intestinal bacteria may be associated with the development and progression of PD. Further studies are required to prove its beneficial effect.

## Introduction

Parkinson’s disease (PD) is a common neurodegenerative disorder, which is characterized by muscular rigidity, bradykinesia, resting tremor, impairment of postural reflex, and non-motor symptoms including dementia. Pathological hallmark of PD is abnormally aggregated α-synuclein protein (Lewy body) in dopaminergic neurons in substantia nigra in the midbrain [1]. The mechanisms underlying abnormal aggregation of α-synuclein have not been fully elucidated. Braak *et al*. observed in autopsies of idiopathic PD patients and healthy elderly people that α-synuclein pathology begins in the dorsal nucleus of vagus nerve, and ascends to raphe nuclei, locus ceruleus, substantia nigra, and cerebral cortex [1]. In 44.6% of PD patients, constipation starts on average 18.1 years before the onset of motor symptoms [2]. Similarly, rapid eye movement (REM) sleep behavior disorder and depression precede the onset of motor symptoms approximately 10 and 5 years, which represent abnormalities in Raphe nucleus and locus ceruleus, respectively [3]. Truncal vagotomy for peptic ulcer in the past markedly decreases a risk for developing PD in Denmark [4] and Sweden [5]. Accumulating evidence supports the notion that abnormal α-synuclein first aggregates in the neural plexus in the gastrointestinal tract, and ascends into the central nervous system.

In PD, intestinal permeability is increased, and its degree is positively correlated with intestinal staining for (i) *Escherichia coli*, (ii) nitrotyrosine, a marker for protein oxidation, and (iii) α-synuclein [6]. Similarly, in a mouse model, hyperpermeability of large intestine and abnormal aggregation of phosphorylated α-synuclein in large intestine are induced by intraperitoneal injection of lipopolysaccharide [7]. Additionally, the effect of intestinal microbiota on PD is underscored by the worsening of PD in a mouse model by transplantation of intestinal microbiota of PD patients [8]. Oxidative stress produced by macrophages in the luminal wall due to a hyperpermeabilized intestinal wall may account for the accumulation of α-synuclein in the intestinal mucosa. As the intestinal microbiota is likely to have a marked effect on the hyperpermeability-induced oxidative stress, the intestinal microbiota may be causally associated with α-synuclein pathology in the enteric nervous system in PD.

Gut microbiota in PD patients have been recently reported by eight groups including us [9–16]. Scheperjans *et al*. compared gut microbiota of 72 PD patients and 72 control subjects by analyzing 16S ribosomal RNA genes. They found that the relative abundance of *Enterobacteriaceae* was positively associated with the severity of postural instability and gait difficulty [9]. Keshavarzian *et al*. analyzed gut microbiota in 38 PD patients and 34 healthy controls. They observed that anti-inflammatory butyrate-producing bacteria from the genera *Blautia*, *Coprococcus*, and *Roseburia* were low in PD patients [10]. Unger *et al*. analyzed fecal concentrations of small chain fatty acids (SCFA) and 9 bacterial phyla/groups by qRT-PCR in 34 PD patients and 34 age-matched controls. They found that SCFA concentrations were low in PD. They also observed that *Enterobacteriaceae* were increased and *Prevotella* were decreased in PD [12], as was observed by Scheperjans *et al*. in Finland [9].

We analyzed 45 PD patients and 34 healthy cohabitants by quantitative RT-PCR of 16S or 23S rRNA of 19 fecal bacterial groups/genera/species [11]. A two-year follow-up study later revealed that a low count of *Bifidobacterium* at year 0 was associated with worsening of PD in two years [17]. We also found that fecal counts of putative hydrogen-producing bacteria were low in PD patients [11]. Among the six fecal bacterial groups/genera/species that were predicted to produce hydrogen, *Bacteroides fragilis* group, *Clostridium perfringens*, *Pseudomonas* possess hydrogenases to produce hydrogen [18]. Similarly, as most strains in *Enterobacteriaceae*, *Blautia* and *Clostridium* produce hydrogen, we assumed that *Enterobacteriaceae*, *Blautia coccoides* group, and *Clostridium leptum* subgroup that we analyzed by qRT-PCR produce hydrogen, but hydrogen-productivity of each bacterium remains to be experimentally determined. Similarly, hydrogen-productivity of the remaining 12 bacteria that we analyzed by qRT-PCR remains unknown.

We reported that hydrogen water prevents the development and progression of PD in a rat model [19]. Similarly, hydrogen in drinking water reduces dopaminergic neuronal loss in the 1-methyl-4-phenyl-1,2,3,6-tetrahydropyridine (MPTP)-induced mouse model of PD [20]. Furthermore, drinking hydrogen water significantly suppresses progression of PD evaluated by Unified Parkinson's Disease Rating Scale (UPDRS) in a randomized placebo-control study of 19 PD patients [21]. A large amount of hydrogen is produced by intestinal anaerobic bacteria in human and rodents [22, 23]. The effects of hydrogen produced by intestinal bacteria are reported in a Concanavarin A (ConA)-induced mouse hepatitis model [24]. They showed that suppression of intestinal microbiota by antibiotics increased the severity of ConA-induced hepatitis, while supplementation of hydrogen-producing *E*. *coli*, but not hydrogen-deficient mutant *E*. *coli*, ameliorated the ConA-induced hepatitis.

In order to examine the roles of hydrogen-producing intestinal bacteria in PD, we individually cultured six bacterial strains representing six most prevalent intestinal bacterial groups/genera/species, and measured the amounts of hydrogen produced by these bacteria. We also analyzed hydrogen production by *Lactobacillus*, the count of which was higher than controls in PD [11].

## Materials and methods

### Bacterial strains

*Blautia coccoides* (JCM 1395) was provided by Japan Collection of Microorganisms, RIKEN BRC, which is participating in the National BioResource Project of the MEXT, Japan. *Clostridium leptum* (ATCC 29065), *Bacteroides fragilis* (ATCC 25285), *Bifidobacterium pseudocatenulatum* (ATCC 27919), *Atopobium parvulum* (ATCC 33793), and *Lactobacillus casei* (ATCC 334) were purchased from ATCC. *Escherichia coli* (W3110) was kindly provided by Dr. Akira Okamoto at the Aichi University of Education.

### Medium

We made culture medium for each bacterium according to the protocols by DSMZ (Table 1). *Blautia coccoides* JCM 1395 and *B*. *fragilis* ATCC 25285 were revived in modified ATCC 1490. The constituents of each medium are shown in S1 Table. The experiments were carried out in a 31-mL screw lip test tube sealed with a butyl rubber stopper and a crimped screw cap with a hole (Sanshin Industrial Co. Ltd.). The test tube contained 5 mL of liquid medium. The headspace in the test tube was flushed with 100% N_2_ gas for DSMZ 58, DSMZ 104, ATCC 416, ATCC 1490 and LB, or with an 8:2 mixture of N_2_ and CO_2_ gas for DSMZ 104c, and the test tube was autoclaved.

**Table 1:** Bacterial strains used for quantification of hydrogen production and the culture medium.

**Genera**	**Strain**	**Medium**^**a**^
*Blautia*	*Blautia (Clostridium) coccoides* (RIKEN BRC JCM 1395)	ATCC 1490
*Clostridium*	*Clostridium leptum* (ATCC 29065)	DSMZ 104c
*Bacteroides*	*Bacteroides fragilis* (ATCC 25285)	ATCC 1490
*Bifidobacterium*	*Bifidobacterium pseudocatenulatum* (ATCC 27919)	DSMZ 58
*Atopobium*	*Atopobium parvulum* (ATCC 33793)	DSMZ 104
*Lactobacillus*	*Lactobacillus casei* (ATCC 334)	ATCC 416
*Escherichia*	*Escherichia coli* (W3110)	LB

^**a**^The constituents of culture medium are indicated in S1 Table.

Fecal bacterial groups/genera/species analyzed by YIF-Scan, but not cultured in this study, are *Prevotella*, *Clostridium perfrigens*, *Enterococcus*, *Staphylococcus*, and *Pseudomonas*.

### Measurement of hydrogen gas concentration

Hydrogen gas concentration was measured as previously described [25] with partial modifications. In brief, each bacterium was first cultured in 5 ml of bacterial culture medium in a 31-mL screw lip test tube, and 100 μl each was inoculated into three or four test tubes containing 5 or 7 ml culture medium (Table 1) followed by static incubation at 37°C under an anaerobic condition.

Bacterial growth was monitored by measuring the absorbance at 600 nm with Miniphoto 518R (Taitec Corp.) every 0.5 to 1.0 hours. The numbers of bacteria were counted at four or more points over a course of culture using Bacteria Counter (Sunlead Glass Corp.) to enable estimation of the bacterial counts from the 600-nm absorbance. A fraction of the headspace gas (100 μl) was intermittently sampled using a gas-tight syringe, and the concentration of hydrogen was measured by a GC-7A gas chromatography equipped with thermal conductivity detector (TCD) (Shimadzu Scientific Instruments, Inc.). For *Blautia coccoides* JCM 1395 and *B*. *fragilis* ATCC 25285, the concentration of hydrogen was measured by a gas chromatography (EAG analyzer GS-23, SensorTec Co. Ltd.) in a high biosafety laboratory. We confirmed that GC-7A with TCD and EAG analyzer GS-23 gave similar values by analyzing hydrogen gas produced by *E*. *coli* W3110. All gas measurements were performed at room temperature.

### Manual annotation of hydrogenase genes in representative human gut strains

Six strains cultured in this study were genome sequenced previously. Genome sequence of *Blautia coccoides* JCM 1395 was not available, and we instead used genome sequence of *Blautia coccoides* YL58. We manually annotated hydrogenase genes of these seven strains using HydDB [26] (S2 Table). Four strains (*Blautia coccoides* YL58, *Clostridium leptum* ATCC 29065, *Bacteroides fragilis* ATCC 25285, and *Escherichia coli* W3110) carried hydrogenase genes, and three strains (*Bifidobacterium pseudocatenulatum* ATCC 27919, *Atopobium parvulum* ATCC 33793, and *Lactobacillus casei* ATCC 334) did not.

Hydrogenase genes in phylogenetic relatives of the four hydrogenase-carrying strains were similarly annotated according to a report by Wolf and colleagues [27] (S3 Table). Phylogenetic relatives of *C*. *leptum* ATCC 29065, *B*. *fragilis* ATCC 25285, *E*. *coli* W3110 were identified using (i) our previous taxonomic coverage analysis [28], which used the same primers as YIF-Scan [11], and (ii) 16S rRNA-based phylogenetic tree of type strains from The All-Species Living Tree Project database (https://www.arb-silva.de/fileadmin/silva_databases/living_tree/LTP_release_128/LTPs128_SSU/LTPs128_SSU_tree.pdf) [29]. Phylogenetic relatives of *B*. *coccoides* JCM 1395 were identified according to a previously reported collation of *B*. *coccoides* group [30].

### Statistical analysis

All analyses were performed with Prism 6 (GraphPad Software Inc.). To compare hydrogen productions by seven bacterial groups/genera/species, one-way ANOVA and the Tukey-Kramer posthoc test were used. To compare hydrogen productions in PD and controls, Student unpaired *t*-test was used. Statistical significance was considered when *p*-value was less than 0.05.

## Results

**Fig 1:**
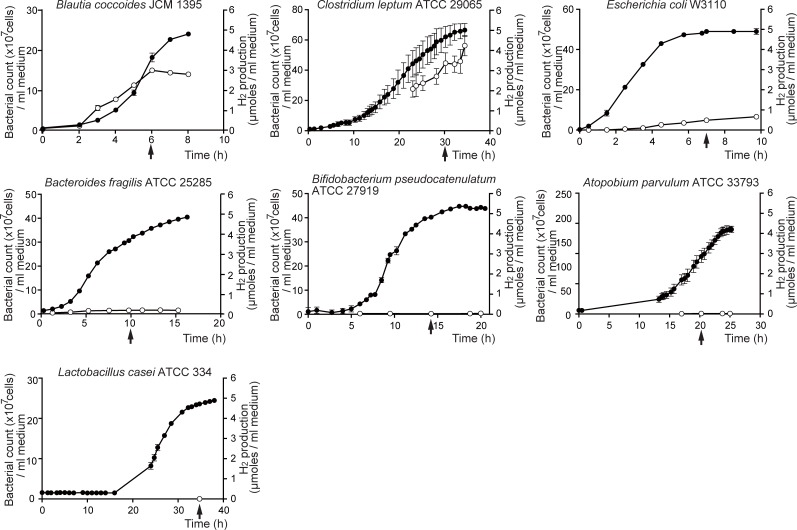
Temporal profiles of the number of bacteria (closed circles) and hydrogen production (open circles). The numbers of bacteria per ml culture medium are plotted on the left axis (closed circles). The hydrogen concentration was quantified by a gas chromatography, and the number of hydrogen molecules per ml culture medium was calculated and plotted on the right axis (open circles). Arrows point to the transition between the growth and stationary phases when the hydrogen concentration accumulated in the gaseous phase was used to calculate the hydrogen production. Note that scales on the right vertical axes are identical, whereas scales on the left vertical axes and on the horizontal axes are different. Mean and SD are indicated (*n* = 3 or 4 culture tubes).

Among the twelve fecal bacterial groups/genera/species that we previously analyzed by qRT-PCR of 16S or 23S rRNA (YIF-Scan) (Table 2 in [11]), we quantified the amount of hydrogen production of six bacterial strains (*Blautia coccoides* JCM 1395, *Clostridium leptum* ATCC 29065, *Bacteroides fragilis* ATCC 25285, *Escherichia coli* W3110, *Bifidobacterium pseudocatenulatum* ATCC 27919 and *Atopobium parvulum* ATCC 33793) representing six most dominant bacterial groups/genera/species according to YIF-Scan (*Blautia coccoides* group, *Clostridium leptum* subgroup, *Bacteroides fragilis* group, *Enterobacteriaceae*, *Bifidobacterium*, and *Atopobium* cluster), respectively. These six prevalent bacterial groups/genera/species constitute 71.3 ± 9.4% (mean and SD) of total fecal bacteria according to the estimation by hybridization with a generic probe Eub338 [31]. The total number of the other six bacterial groups/genera/species in YIF-Scan was 1051- and 223-fold lower than the total number of six prevalent bacterial groups/genera/species in controls and PD patients, respectively [11]. However, as the count of *Lactobacillus* was significantly higher in PD [11], we analyzed hydrogen production by a representative strain *Lactobacillus casei* ATCC 334. We thus cultured seven representative strains individually, and quantified temporal profiles of hydrogen productions by each strain (Fig 1).

**Fig 2:**
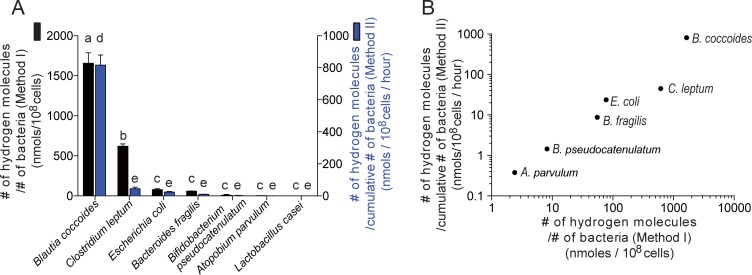
The amount of hydrogen production by seven intestinal bacteria. The amount of hydrogen production was normalized in two ways. Black bars represent the hydrogen production normalized by the number of bacteria at the transition between the growth and stationary phases (arrows in Fig 1) (Method I). Blue bars represent the hydrogen production normalized by the cumulative number of bacteria from time zero to the transition between the growth and stationary phases (arrows in Fig 1) (Method II). The normalized hydrogen productions are indicated in a bar graph on a linear scale **(A)** and in a scattered plot on a logarithmic scale **(B)**. **(A)** Mean and SD are indicated (*n* = 3 or 4 culture tubes). *P* values by one-way ANOVA are < 0.0001 for both Methods I and II. Different symbols in Methods I (a, b, and c) and II (d and e) indicate *p* < 0.05 by Tukey-Kramer posthoc test. **(B)** The Pearson’s correlation coefficient between Methods I and II is 0.950 (*p* = 0.001). Note that *Lactobacillus* is not plotted because the hydrogen production was not detected.

We normalized the amount of hydrogen production in two ways. One was to normalize the hydrogen production by the number of bacteria at the transition between the growth and stationary phases (Method I). The other was to normalize the hydrogen production by the cumulative number of bacteria from time zero to the transition between the growth and stationary phases (Method II). Values normalized by Methods I and II had a Pearson’s correlation coefficient of 0.950 (Table 2 and Fig 2). As hydrogen accumulated during the growth phase was measured at the transition between the growth and stationary phases, Method II was likely to be more dependable than Method I. Indeed, hydrogen production by *Clostridium leptum* ATCC 29065 was 13.7 times lower with Method II compared to Method I (Table 2 and Fig 2). This was likely because *Clostridium leptum* ATCC 29065 grew slower than the other hydrogen-producing bacteria.

**Table 2:** The amounts of hydrogen production by seven bacterial strains representing major intestinal bacterial groups/genera/species.

**Strain**	**# of hydrogen molecules (nmoles)/# of bacteria (cell)**	**# of hydrogen molecules (nmoles)/cumulative # of bacteria (cell) / hour**
*Blautia coccoides* JCM 1395	1.6E-05 ± 1.3E-06	8.1E-06 ± 6.4E-07
*C*. *leptum* ATCC 29065	6.2E-06 ± 2.9E-07	4.5E-07 ± 8.6E-08
*Escherichia coli* W3110	7.8E-07 ± 1.1E-07	2.4E-07 ± 3.3E-08
*B*. *fragilis* ATCC 25285	5.5E-07 ± 4.0E-08	8.8E-08 ± 6.1E-09
*Bifidobacterium pseudocatenulatum* ATCC 27919	8.2E-08 ± 9.7E-08	1.5E-08 ± 1.7E-08
*Atopobium parvulum* ATCC 33793	2.4E-08 ± 4.4E-09	3.8E-09 ± 8.6E-10
*Lactobacillus casei* ATCC 334	0.0E+00 ± 0.0E+00	0.0E+00 ± 0.0E+00

**Fig 3:**
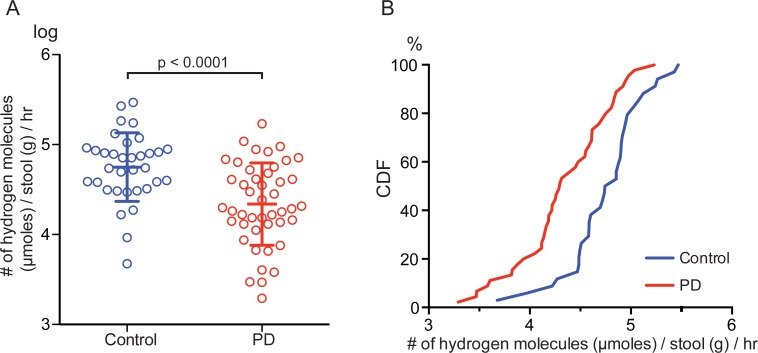
Estimation of the amount of hydrogen production by intestinal microbiota in 45 PD patients and 34 healthy cohabitants that we previously analyzed [11]. The amount of hydrogen production per *g* stool per hour was calculated using the amounts of hydrogen production by seven bacterial strains measured in this study (Method II). **(A)** Geometric distribution plot. Bars represent mean and SD. *P* value was calculated with Student’s *t*-test. **(B)** Cumulative distribution function (CDF) plot.

*Blautia coccoides* JCM 1395 and *Clostridium leptum* ATCC 29065 produced larger amounts of hydrogen gas than the other bacterial strains (Table 2 and Fig 2). *Escherichia coli* W3110 and *Bacteroides fragilis* ATCC 25285 also produced hydrogen, but the amounts were less than those in *Blautia coccoides* JCM 1395 and *Clostridium leptum* ATCC 29065. *Bifidobacterium pseudocatenulatum* ATCC 27919 and *Atopobium parvulum* ATCC 33793 constituted the third group of hydrogen-producing bacteria, and the amounts of hydrogen production were much less compared to the aforementioned four bacteria. *Lactobacillus casei* ATCC 334 produced no detectable hydrogen. Using the hydrogen production by Method II in each bacterium (Table 2), we estimated the amount of hydrogen production by intestinal microbiota in 45 PD patients and 34 healthy cohabitants that we previously analyzed [11]. We multiplied the rate of hydrogen production (nmoles/bacterial cell/hour) (Table 2) by the count of fecal bacterial groups/genera/species estimated by qRT-PCR, and summed up the hydrogen production by seven bacterial strains in each patient or control. The estimated amount of hydrogen production in PD was 2.2-fold lower on average than those in controls (Fig 3). We also estimated intestinal hydrogen production using 16S rRNA amplicon data in Helsinki, Finland (72 PD patients and 72 controls) [9] and Alabama, USA (197 PD patients and 130 controls) [13]. Similar to our cohort, hydrogen production was predicted to be low in PD patients in both countries (S1 Fig).

## Discussion

We quantified the amounts of hydrogen production by seven bacterial strains representing major intestinal bacterial groups/genera/species. There are two possible drawbacks in our study. First, each culture condition was optimized for a specific strain, and was different from the intestinal environment. Simulation of intestinal environment [32, 33] would have yielded more accurate data. Second, we analyzed a single representative strain in each bacterial groups/genera/species. Multiple strains may exist in a single individual, and the dominant strain may be different from individual to individual. Similarly, as the number of genera in intestinal microbiota in human was estimated to be 89 [34], and as not all bacterial species can be cultured *in vitro*, quantification of hydrogen production by all observed strains would be technically unattainable. Despite these limitations, the seven bacterial strains and their taxonomic relatives constituted more than 70% of total fecal bacteria [31].

Among the seven representative bacteria that we analyzed for hydrogen production, three bacterial groups/genera/species (*Blautia coccoides* group, *Clostridium leptum* subgroup, and *Bacteroides fragilis* group) were decreased, whereas *Lactobacillus* was increased in PD [11]. The remaining three bacterial groups/genera/species (*Enterobacteriaceae*, *Bifidobacterium*, and *Atopobium* cluster) were not changed in PD. We found that *Blautia coccoides* JCM 1395 in *Blautia coccoides* group and *Clostridium leptum* ATCC 29065 in *Clostridium leptum* subgroup produced larger amounts of hydrogen gas than the other species. In addition, *Bacteroides fragilis* ATCC 25285 in *Bacteroides fragilis* group constituted the second group of hydrogen production. Thus, the three bacterial strains in three bacterial groups/genera/species that were decreased in PD, produced large amounts of hydrogen. In contrast, *Lactobacillus casei* ATCC 334 in *Lactobacillus*, which was increased in PD, did not produce hydrogen. Lack of hydrogen production in *Lactobacillus casei* has been previously reported [35]. *Lactobacillus casei* produces two lactate molecules from a single glucose molecule (homo lactic acid fermentation) under an anaerobic condition, which does not produce hydrogen.

Hydrogen is either a substrate or a product of hydrogenase. Production of hydrogen is mediated by reduction of protons [36]. The functions of hydrogenases are diversified, and those functions can be predicted by their amino acid sequences [37]. Based on the manual annotation, *Blautia coccoides* JCM 1395, *Clostridium leptum* ATCC 29065, and *Bacteroides fragilis* ATCC 25285 that we cultured have at least one [FeFe]-hydrogenase gene. One of the hydrogenase genes in *B*. *coccoides* JCM 1395 and *C*. *leptum* ATCC 29065 encodes a putative ferredoxin- and NAD-dependent [FeFe]-hydrogenase that is reversibly bifurcates protons from hydrogen to ferredoxin and NAD [38]. In addition, *C*. *leptum* ATCC 29065 and *B*. *fragilis* ATCC 25285 have at least one putative ferredoxin oxidizing [FeFe]-hydrogenase gene. *Escherichia coli* W3110 has putative [NiFe]-hydrogenase coding genes, at least one of which is likely to encode formate hydrogenlyase to metabolize formate and produce hydrogen [39]. Based on the genome information, these four strains have a genetic potential to produce hydrogen. On the other hand, although a very small amount of hydrogen production was observed in *Bifidobacterium pseudocatenulatum* ATCC 27919 and *Atopobium parvulum* ATCC 33793, they do not have hydrogenase genes in the genome, implying the presence of hydrogenase-independent hydrogen production.

According to the phylogenetic distribution of hydrogenase genes (S3 Table), all but one gut strains in close proximity to *B*. *coccoides* JCM 1395, *C*. *leptum* ATCC 29065, and *B*. *fragilis* ATCC 25285 have hydrogenase genes. The presence of [FeFe]-hydrogenase genes and [NiFe]-hydrogenase genes in prokaryotes genomes are relatively rare (approximately 9.1% and 26.7%, respectively [36]), suggesting that hydrogen metabolism is important for these taxa to occupy their niches in human gut. On the other hand, 13 of 21 taxonomic relatives of *E*. *coli* W3110 carried hydrogenases, whereas 8 of them did not, suggesting that the human gut inhabitants of *Enterobacteriaceae* have variable genetic potential of hydrogen production. Although *E*. *coli* W3110 was the third most hydrogen-producing strain, the amount of hydrogen produced by this strain was one magnitude less than those of *B*. *coccoides* JCM 1395 and *C*. *leptum* ATCC 29065. Thus, strain-to-strain variability of hydrogen production in *Enterobacteriaceae* is unlikely to have a major effect on our prediction of intestinal hydrogen production.

A large proportion of hydrogen produced by reduction of ferredoxin, formate and NAD(P)H in hydrogenogens is immediately reoxidized rarely within the same hydrogenogens and mostly by neighboring hydrogenotrophs (acetogens, methanogens, fumarate reducers, and sulfate reducers), which would yield no detectable hydrogen [27, 40]. Thus, the presence of hydrogenase genes does not directly indicate the production of colonic hydrogen pool. According to the healthy Japanese gut metagenome data, almost all of the genes related to methanogenesis are underrepresented compared to the metagenome data in other countries [41]. On the other hand, the genes related to the acetogenesis are overrepresented, and the genes related to the dissimilatory sulfate reduction are similar compared to the metagenome data in other countries [41]. As no shotgun metagenome data are currently available for gut microbiome in Japanese PD patients, we have to perform metagenome-based comparison of the abundance of hydrogenase genes in the future.

We previously reported breath hydrogen concentrations after taking 6 g lactulose in 28 healthy controls and 37 PD patients [42]. A synthetic disaccharide, lactulose, is metabolized by intestinal bacteria and not by human cells. Oral intake of lactulose produces hydrogen as a byproduct [43]. The hydrogen concentrations gradually increased in both controls and PD patients. However, the hydrogen concentrations at 180 min in controls was 1.5 times higher than those in PD, which coincided with our simulation that the amount of hydrogen produced by intestinal microbiota in controls was 1.69 times higher than those in PD (Fig 3). These observations support the notion that hydrogen-producing bacteria are less abundant in PD compared to controls.

Oral administration of lactulose improves chronic portal-systemic encephalopathy [44], atopic dermatitis [45], hyperammonemia [46], and chronic constipation [47]. Although the pharmacological mechanisms of lactulose on human diseases remain to be elucidated, lactulose may exert its effect by increasing hydrogen production by intestinal microbiota [48]. Although we observed no effect of lactulose on 6-OHDA-induced hemi-parkinsonism in rats [42], lactulose may be able to compensate for decreased hydrogen production in PD patients.

Molecular hydrogen reduces hydroxyl radicals *in vitro* [49]. However, hydrogen is unlikely to scavenge hydroxyl radicals in our cells, because the reaction rate constant between hydrogen and hydroxyl radical (4.2 x 10^7^ L•mol^-1^•s^-1^) and the concentration of hydrogen in our bodies (0.005–0.020 mM) [42] are two to three orders of magnitude lower than those of physiological biomolecules (*e*.*g*. amino acids [50], glucose [51], and chloride [52]) that can reduce hydroxyl radicals with the reaction rate constants of 2–10 x 10^9^ L•mol^-1^•s^-1^ and at concentrations of 5–60 mM. Another possible mechanism is that hydrogen induces hormetic responses. First, hydrogen induces mitochondrial unfolded protein response in *C*. *elegans* [53]. Second, multiple reports point to the notion that hydrogen activates the Nrf2 signaling pathway [54–62]. In addition, the effect of hydrogen is not observed in *Nrf2*-knockout mice [55]. Third, we recently reported by meta-analysis of expression arrays that hydrogen induces the heat shock response [63]. The hormetic mechanisms are also inferred from increased reaction oxygen species (ROS) by hydrogen in human [64], rodents [65, 66], and cultured cells [62]. Further studies are required to prove the underlying mechanisms of the effects of hydrogen on PD in mouse [20], rat [19], human [21], and on other diseases [48, 67].

## Supporting information

S1 Fig Estimation of the amount of hydrogen production by intestinal microbiota in 72 PD patients and 72 controls in Helsinki, Finland (A) [9] and 197 PD patients and 130 controls in Alabama, USA (B) [13]. Relative amount of hydrogen production was calculated using the amounts of hydrogen production by seven bacterial strains measured in this study (Method II). In contrast to our YIF-Scan (Fig 3), with which the absolute numbers of living bacteria were estimated by qRT-PCR [11], only relative abundance of each bacterium could be calculated with 16S rRNA amplicon-seq [9, 13]. Geometric distributions normalized to the mean of controls are plotted with mean and SD. *P* value was calculated with Student’s *t*-test.
(EPS)

S1 Table Constituents of culture medium. (DOCX)

S2 Table Manual annotation of hydrogenase genes in seven investigated strains. (DOCX)

S3 Table Phylogenetic distribution of hydrogenase genes in representative human gut strains analyzed by YIF-Scan. (DOCX)

S4 Table Raw data obtained in the current studies. (XLSX)
