# Supplementary material for: Quantification of hydrogen production by intestinal bacteria that are specifically dysregulated in Parkinson's disease
Source: PLoS One. 2018 Dec 26;13(12):e0208313. doi: 10.1371/journal.pone.0208313 (PMC6306167; doi:10.1371/journal.pone.0208313)
Supplement: S1 Table — (DOCX) [file pone.0208313.s002.docx]

**S1 Table. Constituents of culture medium**

| Medium | Constituents |
| --- | --- |
| Modified 1490  (ATCC) | Beef Extract Desiccated 10 g, Trypticase Peptone 30 g, Yeast extract 5 g, K_2_HPO_4_ 5 g, 0.2% Resazurin 0.5 ml, L-cysteine-HCl·H_2_O 0.5 g, Salt solution (see below) 40 ml, Hemin solution (see below) 10 ml, Vitamin K_1_ solution (see below) 0.2 ml, Distilled water 1 L |
| 104c  (DSMZ) | Trypticase peptone 5 g, Peptone from meat (pepsin-digested) 5 g, Yeast extract 10 g, Na-resazurin solution (0.1% w/v) 0.5 mL, Salt solution (see below) 40 ml, L-cysteine-HCl·H_2_O 0.5 g, Na_2_CO_3_ 2.5 g, D-Glucose 5 g, distilled water 1 L |
| LB | Tryptone 10 g, Yeast extract 5 g, NaCl 5 g, Distilled water 1 L |
| 58  (DSMZ) | Casein peptone, tryptic digest 10 g, Yeast extract 5 g, Meat extract 5 g, Bacto Soytone 5 g, glucose 10 g, K_2_HPO_4_ 2 g, MgSO_4_·7H_2_O 0.2 g, MnSO_4_·H_2_O 0.05 g, Tween 80 1 ml, NaCl 5 g, L-cysteine-HCl·H_2_O 0.5 g, salt solution (see below) 40 ml, Resazurin (25 mg / 100 ml) 4 ml, Distilled water 950 ml |
| 104  (DSMZ) | Trypticase peptone 5 g, Peptone 5 g, Yeast extract 10 g, Beef extract 5 g, Glucose 5 g, K_2_HPO_4_ 2 g, Tween 80 1 ml, L-cysteine-HCl·H_2_O 0.5 g, Resazurin 1 mg, salt solution (see below) 40 ml, Hemin solution (see below) 10 ml, Vitamin K_1_ solution (see below) 0.2 ml, distilled water 950 ml |
| 416  (ATCC) | Lactobacilli MRS Broth (BD 288130) 55 g, distilled water 1 L |
| Salt solution | CaCl_2_·2H_2_O 0.25 g, MgSO_4_·7H_2_O 0.5 g, K_2_HPO_4_ 1g, KH_2_PO_4_ 1 g, NaHCO3 10 g, NaCl 2 g, Distilled water 1 L |
| Hemin solution | Hemin 50 mg, 1N NaOH 1 ml, Distilled water 99 ml |
| Vitamin K_1_ solution | Vitamin K_1_ 0.1 ml, 95 % Ethanol 20 ml |
