# Supplementary material for: Quantification of hydrogen production by intestinal bacteria that are specifically dysregulated in Parkinson's disease
Source: PLoS One. 2018 Dec 26;13(12):e0208313. doi: 10.1371/journal.pone.0208313 (PMC6306167; doi:10.1371/journal.pone.0208313)
Supplement: S2 Table — (DOCX) [file pone.0208313.s003.docx]

**S2 Table. Manual annotation of hydrogenase genes in seven investigated strains**

| Strain name | NCBI Genome URL | RefSeq protein ID | Hydrogenase group | HydDB annotation {Sondergaard, 2016 #61} |
| --- | --- | --- | --- | --- |
| *Blautia coccoides* JCM 1395 | https://www.ncbi.nlm.nih.gov/nuccore/NZ_CP022713.1 | WP_065541253.1 | [FeFe] Group A3 | Bifurcating |
| *Clostridium leptum* ATCC 29065 | https://www.ncbi.nlm.nih.gov/nuccore/ABCB00000000.2 | WP_003534157.1  WP_003530568.1  WP_003532170.1  WP_003534146.1 | [FeFe] Group A3  [FeFe] Group B  [FeFe] Group B  [FeFe] Group C | Bifurcating  Evolving  Evolving  Sensory |
| *Bacteroides fragilis* ATCC 25285 | https://www.ncbi.nlm.nih.gov/nuccore/NC_003228.3 | WP_010993508.1 | [FeFe] Group B | Evolving |
| *Bifidobacterium pseudocatenulatum* ATCC 27919 | https://www.ncbi.nlm.nih.gov/nuccore/NZ_AP012330.1 | - | - | - |
| *Atopobium parvulum* ATCC 33793 | https://www.ncbi.nlm.nih.gov/nuccore/NC_013203.1 | - | - | - |
| *Lactobacillus casei* ATCC 334 | https://www.ncbi.nlm.nih.gov/nuccore/NC_008526.1 | - | - | - |
| *Escherichia coli* W3110 | https://www.ncbi.nlm.nih.gov/nuccore/NC_007779.1 | WP_000083065.1  WP_000107384.1  WP_001288122.1  WP_001102321.1 | [NiFe] Group 1c  [NiFe] Group 1d  [NiFe] Group 4a  [NiFe] Group 4a | Anaerobic Uptake  Aerobic Uptake  Evolving  Evolving |

Hydrogenase genes were unlikely to exist in 3 strains, and are indicated by hyphens. Note that *Lactobacillus* produced no hydrogen, and *Bifidobacterium* and *Atopobium* produced marginal amounts of hydrogen.
