# Supplementary material for: Quantification of hydrogen production by intestinal bacteria that are specifically dysregulated in Parkinson's disease
Source: PLoS One. 2018 Dec 26;13(12):e0208313. doi: 10.1371/journal.pone.0208313 (PMC6306167; doi:10.1371/journal.pone.0208313)
Supplement: S3 Table — (DOCX) [file pone.0208313.s004.docx]

**S3 Table. Phylogenetic distribution of hydrogenase genes in representative human gut strains analyzed by YIF-Scan**

| **Strain name** | **Name in YIF-Scan** | **[FeFe]** | **[NiFe]** | **[Fe]** | **Notes** |
| --- | --- | --- | --- | --- | --- |
| *Eubacterium ventriosum* ATCC 27560 | *B. coccoides group* | + | - | - | [FeFe] only |
| *Eubacterium ramulus* VPI C6-27, ATCC 29099 | *B. coccoides* group | + | - | - | [FeFe] only |
| *Roseburia intestinalis* XB6B4 | *B. coccoides group* | + | - | - | [FeFe] only |
| *Clostridium symbiosum* ATCC 14940 | *B. coccoides group* | + | - | - | [FeFe] only |
| *Clostridium bolteae* ATCC BAA-613 | *B. coccoides group* | + | - | - | [FeFe] only |
| *Clostridium clostridioforme* WAL-7855 | *B. coccoides group* | + | - | - | [FeFe] only |
| *Clostridium hathewayi* DSM 13479 | *B. coccoides group* | + | - | - | [FeFe] only |
| *Ruminococcus lactaris* ATCC 29176 | *B. coccoides group* | + | - | - | [FeFe] only |
| *Ruminococcus torques* ATCC 27756 | *B. coccoides group* | + | - | - | [FeFe] only |
| *Dorea formicigenerans* 4_6_53AFAA | *B. coccoides group* | + | - | - | [FeFe] only |
| *Clostridium scindens* ATCC 35704 | *B. coccoides group* | + | - | - | [FeFe] only |
| *Blautia hansenii* VPI C7-24, DSM 20583 | *B. coccoides group* | + | - | - | [FeFe] only |
| *Blautia (Ruminococcus) productus* | *B. coccoides group* | + | - | - | [FeFe] only |
| *Blautia (Clostridium) coccoides* YL58 | *B. coccoides group* | + | - | - | [FeFe] only |
| *Ruminococcus gnavus* ATCC 29149 | *B. coccoides group* | + | - | - | [FeFe] only |
| *Clostridium nexile* DSM 1787 | *B. coccoides group* | + | - | - | [FeFe] only |
| *Eubacterium rectale* DSM 17629 | *B. coccoides group* | + | - | - | [FeFe] only |
| *Blautia (Ruminococcus) hydrogenotrophica* DSM 10507 | *B. coccoides group* | + | - | - | [FeFe] only |
| *Ruminococcus obeum* ATCC 29174 | *B. coccoides group* | + | - | - | [FeFe] only |
| *Butyrivibrio crossotus* DSM 2876 | *B. coccoides group* | + | - | - | [FeFe] only |
| *Eubacterium hallii* DSM 3353 | *B. coccoides group* | + | - | - | [FeFe] only |
| *Cedecea davisae* 005, DSM 4568 | *Enterobacteriaceae* | - | - | - | None |
| *Citrobacter freundii* 4_7_47CFAA | *Enterobacteriaceae* | - | + | - | [NiFe] only |
| *Citrobacter* sp. 30_2 | *Enterobacteriaceae* | - | + | - | [NiFe] only |
| *Citrobacter youngae* ATCC 29220 | *Enterobacteriaceae* | - | + | - | [NiFe] only |
| *Edwardsiella tarda* ATCC 23685 | *Enterobacteriaceae* | - | + | - | [NiFe] only |
| *Enterobacter cancerogenus* ATCC 35316 | *Enterobacteriaceae* | - | - | - | None |
| Enterobacter cloacae cloacae NCTC 9394 | *Enterobacteriaceae* | - | - | - | None |
| Enterobacteriaceae bacterium 9_2_54FAA | *Enterobacteriaceae* | - | + | - | [NiFe] only |
| *^a^Escherichia coli* W3110 | *Enterobacteriaceae* | - | + | - | [NiFe] only |
| *Escherichia coli* ATCC 35469 | *Enterobacteriaceae* | - | + | - | [NiFe] only |
| *Escherichia coli* D9 | *Enterobacteriaceae* | - | + | - | [NiFe] only |
| *Escherichia* sp. 1_1_43 | *Enterobacteriaceae* | - | + | - | [NiFe] only |
| *Escherichia* sp. 3_2_53FAA | *Enterobacteriaceae* | - | + | - | [NiFe] only |
| *Escherichia* sp. 4_1_40B | *Enterobacteriaceae* | - | + | - | [NiFe] only |
| *Hafnia alvei* ATCC 51873 | *Enterobacteriaceae* | - | + | - | [NiFe] only |
| *Klebsiella oxytoca* KA-2 | *Enterobacteriaceae* | - | - | - | None |
| *Klebsiella pneumoniae* WGLW5 | *Enterobacteriaceae* | - | - | - | None |
| *Klebsiella* sp. 1_1_55 | *Enterobacteriaceae* | - | - | - | None |
| *Klebsiella* sp. 4_1_44FAA | *Enterobacteriaceae* | - | - | - | None |
| *Klebsiella* sp. MS 92-3 | *Enterobacteriaceae* | - | - | - | None |
| *Yokenella regensburgei* ATCC 43003 | *Enterobacteriaceae* | - | + | - | [NiFe] only |
| *^a^Clostridium leptum* ATCC 29065 | *C. leptum* subgroup | + | - | - | [FeFe] only |
| *Ruminococcus bromii* L2-63 | *C. leptum* subgroup | - | - | - | None |
| *Subdoligranulum variabile* DSM 15176 | *C. leptum* subgroup | + | - | - | [FeFe] only |
| *Faecalibacterium cf. prausnitzii* KLE1255 | *C. leptum* subgroup | + | - | - | [FeFe] only |
| *Anaerotruncus colihominis* DSM 17241 | *C. leptum* subgroup | + | - | - | [FeFe] only |
| *Clostridium methylpentosum* R2, DSM 5476 | *C. leptum* subgroup | + | - | - | [FeFe] only |
| *Eubacterium siraeum* DSM 15702 | *C. leptum* subgroup | + | - | - | [FeFe] only |
| *Flavonifractor plautii* ATCC 29863 | *C. leptum* subgroup | + | - | - | [FeFe] only |
| *Butyricicoccus pullicaecorum* 1.2 | *C. leptum* subgroup | + | - | - | [FeFe] only |
| *Bacteroides caccae* ATCC 43185 | *B. fragilis* group | + | - | - | [FeFe] only |
| *Bacteroides cellulosilyticus* DSM 14838 | *B. fragilis* group | + | - | - | [FeFe] only |
| *Bacteroides clarus* YIT 12056 | *B. fragilis* group | + | - | - | [FeFe] only |
| *Bacteroides coprocola* M16, DSM 17136 | *B. fragilis* group | + | - | - | [FeFe] only |
| *Bacteroides coprophilus* DSM 18228, JCM 13818 | *B. fragilis* group | + | - | - | [FeFe] only |
| *Bacteroides dorei* DSM 17855 | *B. fragilis* group | + | - | - | [FeFe] only |
| *Bacteroides eggerthii* 1_2_48FAA | *B. fragilis* group | + | - | - | [FeFe] only |
| *Bacteroides finegoldii* DSM 17565 | *B. fragilis* group | + | - | - | [FeFe] only |
| *Bacteroides fluxus* YIT 12057 | *B. fragilis* group | + | - | - | [FeFe] only |
| *^a^Bacteroides fragilis* ATCC 25285 | *B. fragilis* group | + | - | - | [FeFe] only |
| *Bacteroides fragilis* 3_1_12 | *B. fragilis* group | + | - | - | [FeFe] only |
| *Bacteroides intestinalis* 341, DSM 17393 | *B. fragilis* group | + | - | - | [FeFe] only |
| *Bacteroides oleiciplenus* YIT 12058 | *B. fragilis* group | + | - | - | [FeFe] only |
| *Bacteroides ovatus* ATCC 8483 | *B. fragilis* group | + | - | - | [FeFe] only |
| *Bacteroides pectinophilus* ATCC 43243 | *B. fragilis* group | + | - | - | [FeFe] only |
| *Bacteroides plebeius* M12, DSM 17135 | *B. fragilis* group | + | - | - | [FeFe] only |
| *Bacteroides salyersiae* WAL 10018, DSM 18765, JCM 12988 | *B. fragilis* group | + | - | - | [FeFe] only |
| *Bacteroides* sp. 1_1_14 | *B. fragilis* group | + | - | - | [FeFe] only |
| *Bacteroides* sp. 1_1_30 | *B. fragilis* group | + | - | - | [FeFe] only |
| *Bacteroides* sp. 1_1_6 | *B. fragilis* group | + | - | - | [FeFe] only |
| *Bacteroides* sp. 2_1_16 | *B. fragilis* group | + | - | - | [FeFe] only |
| *Bacteroides* sp. 2_1_22 | *B. fragilis* group | + | - | - | [FeFe] only |
| *Bacteroides* sp. 2_1_33B | *B. fragilis* group | + | + | - | [FeFe], [NiFe] |
| *Bacteroides* sp. 2_1_56FAA | *B. fragilis* group | + | - | - | [FeFe] only |
| Bacteroides sp. 2_2_4 | *B. fragilis* group | + | - | - | [FeFe] only |
| *Bacteroides* sp. 20_3 | *B. fragilis* group | + | + | - | [FeFe], [NiFe] |
| *Bacteroides* sp. 3_1_13 | *B. fragilis* group | + | - | - | [FeFe] only |
| *Bacteroides* sp. 3_1_19 | *B. fragilis* group | + | + | - | [FeFe], [NiFe] |
| *Bacteroides* sp. 3_1_23 | *B. fragilis* group | + | - | - | [FeFe] only |
| *Bacteroides* sp. 3_1_33FAA | *B. fragilis* group | + | - | - | [FeFe] only |
| *Bacteroides* sp. 3_1_40A | *B. fragilis* group | + | - | - | [FeFe] only |
| *Bacteroides* sp. 3_2_5 | *B. fragilis* group | + | - | - | [FeFe] only |
| *Bacteroides* sp. 4_1_36 | *B. fragilis* group | + | - | - | [FeFe] only |
| *Bacteroides* sp. 4_3_47FAA | *B. fragilis* group | + | - | - | [FeFe] only |
| *Bacteroides* sp. 9_1_42FAA | *B. fragilis* group | + | - | - | [FeFe] only |
| *Bacteroides* sp. D1 | *B. fragilis* group | + | - | - | [FeFe] only |
| *Bacteroides* sp. D2 | *B. fragilis* group | + | - | - | [FeFe] only |
| *Bacteroides* sp. D20 | *B. fragilis* group | + | - | - | [FeFe] only |
| *Bacteroides* sp. D22 | *B. fragilis* group | + | - | - | [FeFe] only |
| *Bacteroides* sp. HPS0048 | *B. fragilis* group | + | - | - | [FeFe] only |
| *Bacteroides stercoris* ATCC 43183 | *B. fragilis* group | + | - | - | [FeFe] only |
| *Bacteroides uniformis* ATCC 8492 | *B. fragilis* group | + | - | - | [FeFe] only |
| *Bacteroides vulgatus* PC510 | *B. fragilis* group | + | - | - | [FeFe] only |
| *Bacteroides xylanisolvens* XB1A | *B. fragilis* group | + | - | - | [FeFe] only |

Presence and absence of specific hydrogenase gene are indicated by + and -, respectively.

*^a^*Four strains producing hydrogen, which were cultured in our current study.
